# Radiation capture and conversion efficiencies of *Miscanthus sacchariflorus*,* M. sinensis* and their naturally occurring hybrid *M*. × *giganteus*

**DOI:** 10.1111/gcbb.12331

**Published:** 2016-02-26

**Authors:** Christopher Lyndon Davey, Laurence Edmund Jones, Michael Squance, Sarah Jane Purdy, Anne Louise Maddison, Jennifer Cunniff, Iain Donnison, John Clifton‐Brown

**Affiliations:** ^1^ Institute of Biological, Environmental and Rural Sciences (IBERS)Aberystwyth UniversityGogerddanAberystwyth, CeredigionSY23 3EEUK; ^2^ Rothamsted ResearchHarpenden, HertfordshireAL5 2JQUK

**Keywords:** diversity, extinction coefficient, *Miscanthus*, radiation‐use efficiency, sensitivity analysis, yield

## Abstract

*Miscanthus* is a rhizomatous C4 grass of great interest as a biofuel crop because it has the potential to produce high yields over a wide geographical area with low agricultural inputs on marginal land less suitable for food production. At the moment, a clonal interspecific hybrid *Miscanthus* × *giganteus* is the most widely cultivated and studied in Europe and the United States, but breeding programmes are developing newer more productive varieties. Here, we quantified the physiological processes relating to whole season yield in a replicated plot trial in Wales, UK. Light capture and conversion efficiency were parameterized for four carefully selected genotypes (*M. sinensis*,* M. sacchariflorus* and *Miscanthus* × *giganteus*). Differences in the canopy architecture in mature stands as measured by the extinction coefficient (*k*) were small (0.55–0.65). Sensitivity analysis on a mathematical model of *Miscanthus* was performed to quantify the accumulative intercepted photosynthetically active radiation (iPAR) in the growing season using (i) k, (ii) variation in the thermal responses of leaf expansion rate, (iii) base temperature for degree days and (iv) date start of canopy expansion. A 10% increase in *k* or leaf area per degree day both had a minimal effect on iPAR (3%). Decreasing base temperature from 10 to 9 °C gave an 8% increase in iPAR. If the starting date for canopy expansion was the same as shoot emergence date, then the iPAR increases by 12.5%. In *M*. × *giganteus,* the whole season above ground and total (including below ground) radiation‐use efficiency (RUE) ranged from 45% to 37% higher than the noninterspecific hybrid genotypes. The greater yields in the interspecific hybrid *M. × giganteus* are explained by the higher RUE and not by differences in iPAR or partitioning effects. Studying the mechanisms underlying this complex trait could have wide benefits for both fuel and food production.

## Introduction

*Miscanthus* is a rhizomatous C4 grass of interest as a potential biofuel crop (Visser & Pignatelli, 2001; Hastings *et al*., 2009a; Somerville *et al*., 2010; Zub & Brancourt‐Hulmel, 2010). This is because it has the potential to produce high yields (Clifton‐Brown *et al*., 2001) over a wide geographical area with low agricultural inputs (Beale & Long, 1997; Zub & Brancourt‐Hulmel, 2010) and can be grown on marginal land not cultivated for food production. At the moment, a *Miscanthus* × *giganteus* genotype is the one most often grown and studied in Europe because of its high yields. *M*. × *giganteus* is a naturally occurring hybrid of *M. sinensis* and *M. sacchariflorus* (Greef & Deuter, 1993; Hodkinson & Renvoize, 2001). As *Miscanthus* is an undomesticated plant, there is scope to increase yields over *M*. × *giganteus* and so breeding programmes are ongoing to achieve this by utilizing the considerable phenotypic diversity found across the genotypes (Robson *et al*., 2013). The range of this diversity has not been systematically modelled at the level of descriptions of the physiological processes relating to yield. Nor has the range of the variation been used to inform which features of *Miscanthus* are most amenable to giving increases in yield above those produced by *M*. × *giganteus*.

To address both these issues, a physiologically based model of yield is needed which can be easily parameterized for different genotypes. The range of parameter values can then be used in ‘what if’ simulations using the model to access their impact on yield using *M*. × *giganteus* as a reference. Such a study using different genotypes would only give an indication of the variation in key yield parameters currently expressed in the breeding populations. In addition, genetic segregation and recombination during breeding would be expected to produce further variation especially in a genetically diverse and nondomesticated plant like *Miscanthus* (Hartl & Clark, 2007).

A fundamental model of yield based on the work of Monteith (Monteith, 1977) is as follows:

(1) $$\begin{array}{cc} & \text{Yield}\;=\\ & \;\;\text{Sum}\;\text{incident}\;\;\text{PAR}\;\;\text{over}\;\;\text{current}\;\;\text{growing season}\;\\ & \;\mathrm{length}\;*\text{Proportion}\;\;\text{PAR}\;\text{intercepted}\;\text{*}\;\text{RUE} \end{array}$$

For *Miscanthus,* yield is the above‐ground dry matter at harvest. PAR is photosynthetic active radiation and RUE is the radiation‐use efficiency which quantifies the amount of dry matter created for each MJ of PAR the canopy intercepts.

The proportion of PAR intercepted by the canopy in Eqn (1) has two components. The first is the innate ability of the canopy architecture to capture light: this is quantified by the canopy's extinction coefficient (*k*). The second component is the development of the canopy leaf area index (LAI in m^2^ leaf area m^−2^ ground) over the growing season. This depends on the start date and rate of canopy expansion. Canopy expansion is largely driven by temperature above a base level (*T*_b_) below which the leaf expansion ceases. This affect is quantified by the leaf expansion rate (LER) in LAI °C day^−1^, where the denominator is the degree days above *T*_b_. Thus, there is a complex interaction in yield production of the canopy intercepting the ambient PAR but with the canopy expansion (and hence its ability to intercept light) being driven by temperature. To combine the influences of PAR levels and temperature on yield over a growing season, one needs to convert Eqn (1) to a model which can be stepped through time using real met data. The model could then be made genotype specific using the appropriate values of the parameters *T*_b_, LER, *k* and RUE.

The genotypes investigated in this study include two *M. sinensis* a *M. sacchariflorus* and the hybrid *M*. ×*giganteus*. They were selected based on their large variations in canopy architecture and also variation in flowering time which is known to affect yield (Jensen *et al*., 2013). *Miscanthus sinensis* genotypes (including those used in this trial) tend to flower prolifically, whereas *M. sacchariflorus* does not flower in the field in the United Kingdom and *M*. × *giganteus* only flowers on exceptionally warm years (Jensen *et al*., 2013).

In this study, field data are first used to estimate *T*_b_, LER, *k* and RUE for the four genotypes. This provides an estimate of the cross‐genotype (species) variation that can be expected in these key values that determine light interception and yield. In particular, the impact of the widely different canopy architectures of the plants on light interception (via k) can be accessed. In addition, the RUEs (calculated with the aid of the model in Eqn (1)) can be used to help understand why *M*. × *giganteus* is such a productive genotype. The values of *T*_b_, LER, *k* and RUE are of direct use in reparameterizing complex models of *Miscanthus* to emulate different genotypes, but this would require additional work to access the by‐genotype values for other model parameters. To avoid this, the simpler model in Eqn (1) is used to investigate by simulation the light capturing ability and yield of a potential new hybrid created by incorporating into it the variation in values of *T*_b_, LER, *k* and RUE seen in this study. This is achieved by inserting these values into the model of *M*. × *giganteus* and accessing if this increases its performance. Thus, suggestions on potential breeding targets can also be made.

## Materials and methods

### Plant material and trial configuration

**Figure 1:**
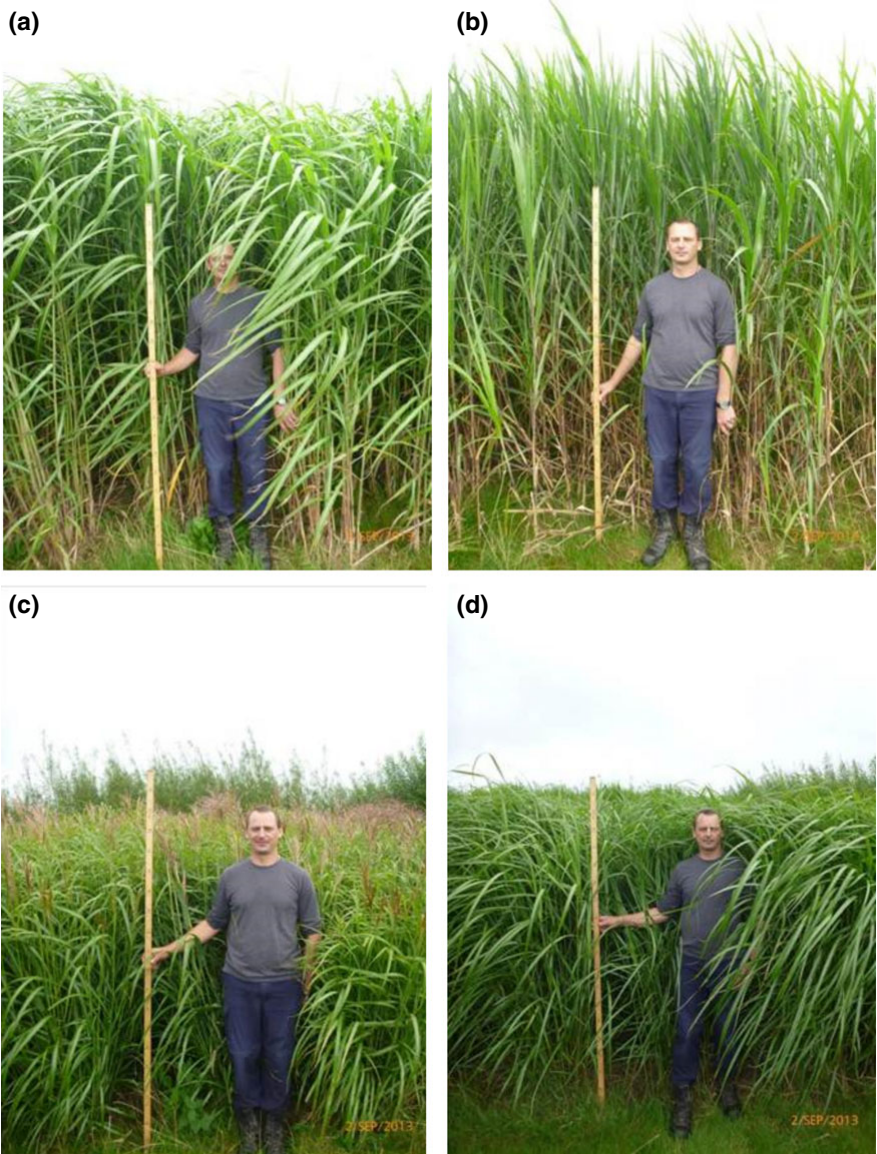
Photographs showing the different canopy architectures of the four genotypes used in the trial (taken in September 2013): (a) Gig‐311, (b) Sac‐5, (c) Sin‐11 and (d) Goliath.

Four genotypes were selected that included the extremes of *Miscanthus* canopy morphology (Fig. 1). Two were *M. sinensis* types: Sin‐11 (also known as Emi‐11) a diploid and Goliath a triploid. Sac‐5 was a tetraploid *M. sacchariflorus* genotype and *M*. × *giganteus* (clone Gig‐311) a triploid hybrid of *M. sinensis* and *M. sacchariflorus*. The trial was situated on former grassland at IBERS on the West Wales (UK) coast (52.4139′N, −4.014′W). It consisted of a randomized block design with four blocks each containing a replicate plot of each genotype. All the plots contained 121 plants at a density of 2 plants m^−2^ and were established using rhizome grown plantlets in May 2009. An automated meteorological station (Campbell Scientific) fitted with a CR1000 data logger at the trial site recorded soil and air temperature, PAR levels and soil moisture content. Additional weather data came from met stations at nearby field sites. A replicated trial was run at Rothamsted Research (Harpenden, Hertfordshire, England, UK) which also carried out all the measurements taken at IBERS except for LAI. Unless otherwise stated, data are for IBERS.

### Emergence

The above‐ground biomass of the *Miscanthus* was removed on 20 February in 2011. Emergence of new buds from the rhizomes was then scored at weekly intervals on four randomly selected (pseudo‐replicate) plants in each plot. Stage ‘NEB’ indicated the presence of at least one newly emerged bud (shoot). There was no frost causing damage to the emerged shoots in 2011. A bud from each plant was then monitored, and its progressive development recorded. Stage ‘FLL’ was scored when its first leaf with a ligule was observed. The plot was designated as being at stage NEB or FLL when two of the four plants reached the given stage.

### Destructive harvests

Destructive harvests were carried out periodically through the growing season starting on the 21 February 2011 (before emergence) until January 2012. A single plant per experimental plot was randomly selected and the above‐ground material cut at 10 cm height. A quadrat was used to demarcate the plant's 0.5 m^2^ ground area and the rhizome completely excavated and then washed. Samples of the above‐ and below‐ground material were dried in a drying oven until constant mass and the total above‐ and below‐ground dry weights were then calculated.

### Leaf area index (LAI) measurements

LAI was estimated from repeat measurements of leaf area on marked stems and from counts of stem numbers m^−2^. Stem counts were repeatedly carried out on at least three randomly selected plants per experimental plot. This was made at weekly or 2‐weekly intervals on the same plants at 60% of the canopy height (Clifton‐Brown *et al*., 2000). This height was chosen as it included all the light capturing leaf area but excluded later emerging stems with small leaf areas from the stem count. Including such stems in the stem counts would artificially elevate the LAI if multiplied by the large leaf areas on the older leaf area measurement stems which formed the canopy itself. The pseudo‐replicate stem counts were averaged to give a single stem number m^−2^ value for each experimental plot on a given day.

On each experimental plot, three plants were picked at random and a single stem from each randomly selected and marked for repeat leaf area measurements. Measurements were made weekly or every 2 weeks. As each leaf unfurled, it was numbered and its length and maximum width were measured until it gained a ligule at which point its area no longer increased. The highest leaf with a ligule in the last measurements was remeasured to ensure its dimensions were unchanged. All leaves were checked and if they had died or badly fragmented, they were given a leaf area of zero. The leaf area measurements ceased before the onset of canopy senescence and so green leaf area and green LAI were estimated.

To convert field leaf dimension data to actual areas, a calibration data set was produced. For each genotype, a single mature stem was selected at random from each replicate experimental plot. Every undamaged leaf was measured as in the field and its real area estimated using image analysis. This was performed twice, once at the beginning of July 2011 to include the small early season leaves before they died. The second set was in mid‐August 2012 so that the very large late season leaves were included. The data for each genotype were combined and plotted as actual leaf area (cm^2^) vs. leaf length * maximum leaf width (cm^2^). Genotypes with significantly different straight line fits to their data were found by ancova using r's lm() function (R Core Team, 2013) as described in Crawley (2007). A 5% significance level was used for all statistical tests in this article.

The slopes of the significantly different straight lines fitted to the leaf area calibration data were then used to convert all the field leaf dimension data to actual leaf areas and hence to leaf areas per stem. On each measurement day, the leaf areas of the pseudo‐replicate stems on each experimental plot were averaged to give a single value of m^2^ leaf area stem^−1^ for that plot. Typically, the stem counts were estimated on different days to the leaf area measurements and so they were adjusted to give the counts on the leaf measurement days using linear interpolation. Once this had been performed, multiplying the experimental plot's average m^2^ leaf area stem^−1^ by the equivalent average stem number m^−2^ ground gave the LAI (m^2^ leaf area m^−2^ ground).

### Canopy PAR transmission measurements and extinction coefficient (*k*) estimations

For each experimental plot, three plants were selected at random for repeat transmission measurements using a SunScan SS1 (Delta T Devices Ltd, Cambridge, UK: www.delta-t.co.uk/) at weekly or 2‐weekly intervals. The instrument consisted of hand‐held device with a 1 metre long probe with 64 diodes that measure PAR intensities and a separate station that measures the PAR incidence on the canopy top. The measurements were made in accordance with the manufacturer's instructions. In particular, all readings were taken within 2 h either side of solar noon and not on rainy or dark cloudy days. Stakes were placed around each plant so that measurements could be made with the probe centred at the plant's middle at ground level. They were situated so that a pair of measurements could be made, one in a south‐westerly and the other in a south‐easterly direction. For each plant on a plot, one probe measurement was taken in each direction and then converted to transmission values by dividing the mean diode PAR readings by the PAR incident on the canopy top. The two transmissions were then averaged to give the plant's transmission. The pseudo‐rep plant transmissions were averaged to give a single value for the plot's transmission. Linear interpolation was then used to find the plot's transmission on the days equivalent to the LAIs. These transmissions were then averaged across the replicate plots to give the mean transmissions for each genotype over time. Loss of transmission data in 2011 meant some additional measurements of transmission, and LAI was made in 2012 to allow the estimation of the canopy extinction coefficient (*k*).

For each genotype, the mean transmission values were plotted against the mean LAIs. For Gig‐311, Sac‐5 and Goliath, the 2011 and 2012 data sets were combined. However, for Sin‐11, the data from the 2 years were very different from each other and therefore treated separately. The *k* values were estimated by fitting the formula transmission = e^−*k* * LAI^ to the data using nonlinear regression with grouped data as described in Ritz & Streibig (2008). Pairs of genotypes were compared using the r nls() function, first using a separate *k* for each genotype and then using a model with the same *k* for both. An *F*‐test on the two fits was then used to determine whether the *k* values were significantly different.

### Estimating the leaf expansion rate (LER, LAI °C day^−1^) values

Initially, the strategy used in Clifton‐Brown *et al*. (2000) to estimate the LER of Gig‐311 was adopted. This was to plot LAI vs. cumulative degree days ( °C days) calculated using a given *T*_b_ value and then to fit the data by linear regression. This was repeated for a range of *T*_b_ values and the fit with the lowest *R* ^2^ gave both the LER from its slope and the optimal *T*_b_. However, during this study, it was found that some of the genotypes had more than one growth phase and that *R* ^2^ was very insensitive to *T*_b_ for the data used here. It was therefore decided to adopt a *T*_b_ of 0 °C as was used by Hastings *et al*. (2009b) for *Miscanthus* modelling.

The two growth patterns that occurred were as follows: (i) a single straight line and (ii) a change in growth rate that could be approximated by two straight lines with a single breakpoint between them. Linear regression using the r lm() function was used for the 1st growth pattern data. Growth pattern 2 was fitted using segmented regression using the r ‘Segmented’ library (Muggeo, 2008) which gave both straight lines and the breakpoint in one fit. Each experimental field plot was fitted separately using the minimal number of lines that gave a reasonable fit to the data so that between‐plot variation could be accessed. In all cases, the LAI and cumulative degree days were set as zero on the actual day that particular experimental plot first produced leaves with ligules (stage FLL) and the late season steady‐state LAIs when shading effects occurred were excluded.

The slopes of the two possible growth phases on the LAI vs. degree day graphs gave the LERs. The replicate LER values for each genotype (one per replicate field plot) were then compared to the other genotypes by anova. This was performed using the r aov() function and the Duncan's multiple range test from the R library ‘Agricolae’ (Mendiburu, 2013). Where the mean genotype LERs were not significantly different, the mean LER across the genotypes was used as the final LER value, otherwise the significantly different genotype mean was used.

For use in simulations, the mean LAI values for Gig‐311 across the four replicate plots were plotted against degree days using the known base temperature of this genotype of 10 °C (Clifton‐Brown *et al*., 2000). The data were fitted to a single straight line as above to give the equivalent LER.

### Simulations

**Figure 2:**
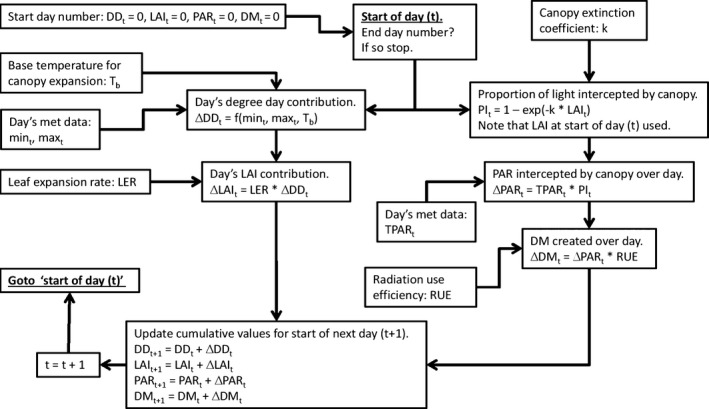
The mathematical model of *Miscanthus* canopy development and yield represented as a flowchart. One cycle around the chart steps the model through one day using that day's met data. ▵: value (change) over given day t. DD: thermal time (degree days in °C days). DM: dry matter (gDM m^−2^ ground). f(min_t_, max_t_, *T*_b_): the formula for calculating DD using the daily min and max air  °C values and the base temperature for canopy expansion (*T*_b_) (Clifton‐Brown *et al*. (2000)). *k*: canopy extinction coefficient (m^2^ ground m^−2^ leaf, i.e. dimensionless). LAI: leaf area index (m^2^ leaf m^−2^ ground, i.e. dimensionless). LER: leaf expansion rate which is the degree day to LAI conversion factor (LAI °C day^−1^, or  °C day^−1^). If a genotype has more than one growth phase, then this value may be switched to a different one once a threshold cumulative DD value has been reached. Max_t_: maximum air temperature on day *t* ( °C). Min_t_: minimum air temperature on day *t* (° C). PAR: photosynthetically active radiation (MJ m^−2^ ground). PI_t_: proportion of the PAR hitting the top of the canopy that is intercepted by the canopy on a given day (dimensionless). RUE: radiation‐use efficiency (gDM MJ ^−1^ intercepted PAR). t (subscript): day number in year. *T*_b_: base temperature for canopy expansion ( °C). TPAR_t_: total PAR hitting the top of the canopy over day *t* (MJ m^−2^ ground).

Simulations were executed by running the model in Fig. 2 in the modelling program Simile (Simulistics, Edinburgh, UK: www.simulistics.co.uk). The model consisted of four coupled ordinary differential equations in which Simile were represented graphically in System Dynamics notation (Haefner, 1996). These equations form the core of the *Miscanthus* model MiscanMod (Clifton‐Brown *et al*., 2000) which can be downloaded from the PLASMO web‐portal (www.plasmo.ed.ac.uk/plasmo). The model was stepped through time in Simile using an Euler numerical integrator with 1‐day time steps using the genotype specific values for the parameters *T*_b_, LER, *k* and RUE derived from the field data. In Simile, the representation of the model and the integration algorithm are kept strictly separate (Muetzelfeldt, 2004), and the version of the model in Fig. 2 is a combination of the model and the Euler integrator.

For the simulations and RUE calculations, it was necessary to confirm that the mean genotype values for the LERs derived from fitting each individual field plot, and the mean breakpoint degree days for those with two growth phases still enabled the model to predict the actual mean LAIs of each genotype. This was performed by running the model parameterized for each genotype: starting with zero LAI and cumulative degree days on the actual stage FLL day and using the 2011 met data to drive the model (see Fig. 2). The simulated LAI values were then plotted with the equivalent real mean genotype LAIs (across the four replicate experimental field plots) against time. As with all the graphs in this article, this was carried out in R using the error bar function from the library ‘Plotrix’ (Lemon, 2006) as required.

Simulation was then used to estimate the LAIs of each genotype over the growing season, including for the missing values just after the canopy started to expand, and using the fitted *k* values, the cumulative PAR on each day could also be found. These simulations were used for two purposes. Firstly, they gave the cumulative PAR values on the days when destructive harvests gave the equivalent plant dry weights so enabling the calculation of the RUEs. Later in the season, the plants achieved a steady‐state LAI which the model fails to predict. Over this time range, the simulated LAIs were replaced by values found from linear interpolation between the real LAIs and the met PAR data then used to increment the cumulative PAR interception accordingly. These adjusted cumulative PAR values were then used in the estimation of the genotype RUEs although the correction made only a few percentage points difference to the cumulated PAR because of the canopy intercepting ‘all’ of the light from quiet early in the growing season.

The second use of simulation was to access the effect of the variation in the fitted parameter values on potential cumulative PAR interception. As Gig‐311 was the genotype to improve over, the effects were investigated by looking at the changes in cumulative PAR that they would produce in Gig‐311 (i.e. in an equivalent new hybrid). Gig‐311 reached steady‐state LAIs by day 230, and as the model does not account for this, the simulations were run from its stage FLL day until day 230. By this stage, all the plants were intercepting light at the same rate (due to their high LAIs) and so the few weeks until the first plants started to senesce wont materially affect the conclusions expressed relative to Gig‐311. For each simulation, a reference run was carried out with Gig‐311 having only its fitted parameter values. Percentage changes in cumulative PAR on day 230 for the additional simulations were expressed relative to the equivalent value for the reference simulation using: 100 (simulation –ref simulation)/ref simulation. The simulations on the variations in fitted *k* and LER used the model parameterized for a *T*_b_ of 0 °C. For Gig‐311, the *T*_b_ is known to be 10 °C, and for simulations of the effect of *T*_b_ changes on the cumulative PAR intercepted, the LER estimated with this base temperature was used. All the simulations used the 2011 met data set.

### Estimating the radiation‐use efficiencies (RUE)

The destructive harvests gave the plant dry matter values on particular days. The simulations above also gave the cumulative PAR interception values for each genotype on the same days up to the 5 September harvest. The destructive harvest before the plants emerged (21 February) was assumed to give the baseline below‐ground biomass with the above‐ground mass as zero because of the removal of the previous year's growth. These values corresponded to zero cumulative light interception by the plants. The slopes of straight lines fitted to plots of dry matter vs. cumulative PAR interception gave the RUEs in g dry matter MJ^−1^ PAR intercepted. For each genotype, three such plots were done with the below‐ground, above‐ground and total dry matter on the *y*‐axis. These gave the below‐ground, above‐ground and total RUEs, respectively. The straight lines were fitted either to each genotype's data by linear regression, or ancova was used to find across genotype values as described above.

## Results

### Climate in 2011 and 2012

*Miscanthus* yield can be affected by drought (Clifton‐Brown *et al*., 2002; Richter *et al*., 2008). There was a period of slightly lowered rainfall in March/April 2011, but the vegetation cover was low and the soil water was at winter levels before it. Monitoring of stem extension rate did not indicate drought effects during the growing seasons.

### Emergence

The days in the year when new buds from the rhizome first appeared above the ground (stage NEB) and when these produced their first leaves with ligules (stage FLL) are recorded in Table 1. Gig‐311 produced its first buds 2 weeks before Sac‐5 which in turn had buds between 1 and 2 weeks before the other two genotypes. Despite the differences in bud emergence day, all the genotypes produced their first leaves with ligules from these buds within 1 week of each other. The buds of Sin‐11 and Goliath emerged and produced ligule leaves within 1 week. In addition, some emergence from buds that over‐wintered above ground and regreening from stems cut during harvesting was also observed.

**Table 1:** The mean day numbers in the year (in 2011) when newly emerged buds from the rhizomes first appeared above ground (stage NEB day) and when the first leaf with a ligule was recorded (stage FLL day)

Genotype	Day newly emerged buds appeared (stage NEB day)	Day first leaf with a ligule observed (stage FLL day)
Gig‐311	76	102
Sac‐5	92	106
Sin‐11	102	109
Goliath	100	107

**Figure 3:**
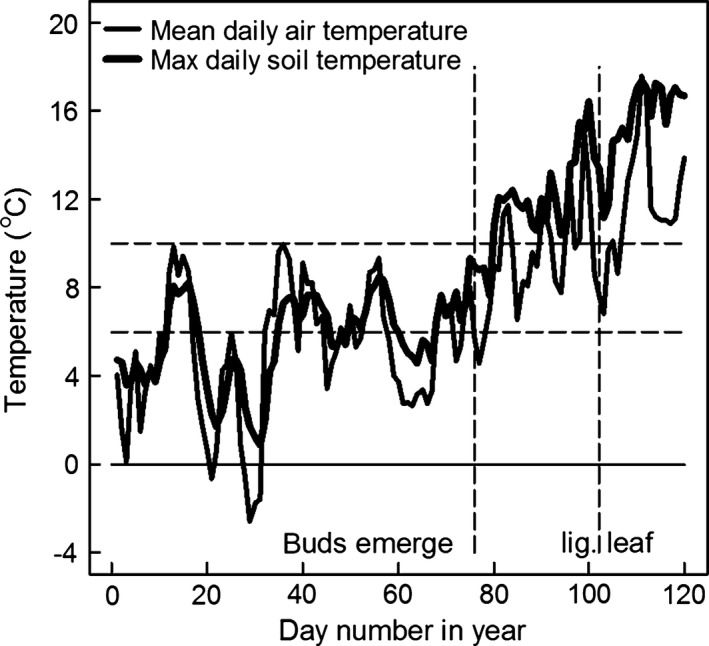
The mean daily air temperature (at 1.5 m) and maximum daily soil temperature (at ‐10 cm) for 2011 up to the dates of the appearance of the first leaves with ligules for the four genotypes. For Gig‐311, new buds from the rhizome emerged above the ground (stage NEB) on day 76 but did not produce the first leaves with ligules (stage FLL) until day 102 (vertical lines on the figure). The horizontal lines on the figure are at 0, 6 and 10 °C. 10 °C is thought to be the base temperature for canopy expansion (*T*_b_) of Gig‐311 whilst 6 °C is the lowest *T*_b_ currently recorded for *Miscanthus*.

Figure 3 shows the mean daily air temperature and maximum daily soil temperature (at 10 cm depth) for the period leading up to bud and ligule leaf emergence in 2011. Marked on the figure are the dates when the buds and first ligule leaves of Gig‐311 appeared. The temperatures at which the buds first appeared above the soil (stage NEB, day 76) were not different from those that had already occurred several times earlier in the year and were below the base temperature (*T*_b_) for canopy expansion of 10 °C. Once the buds emerged, the air temperatures were at times above the 10 °C *T*_b_ value but leaves with ligules were not unfurled from the developing stem (stage FLL) until day 102.

### LAI and canopy development

The field measurements of leaf length and width were converted to real areas using the results from the calibration graph of actual leaf area (cm^2^) vs. leaf length * maximum width (cm^2^). The data gave two significantly different straight line fits both with a zero y‐intercept. Gig‐311 had a conversion factor (slope) of 0.745, whilst the other three genotypes all had factors of 0.684 (*R* ^2^ of the ancova was 0.99).

**Figure 4:**
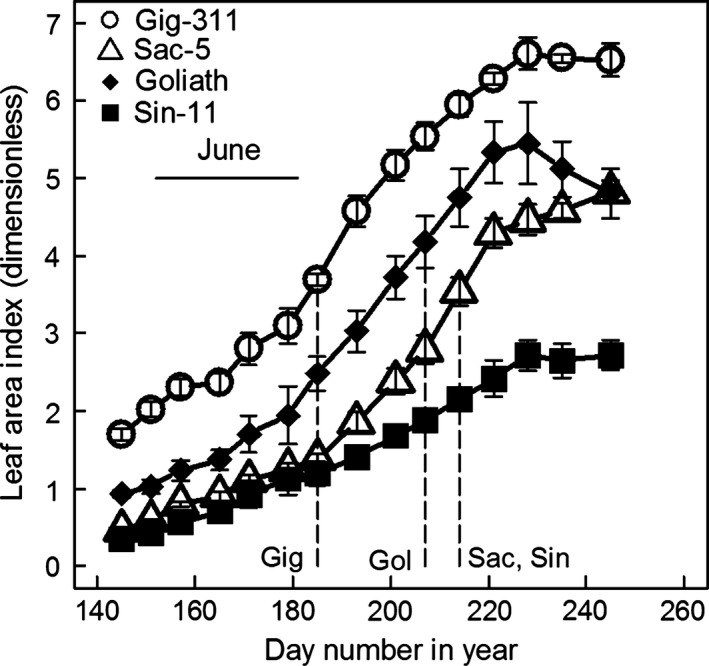
The mean LAIs across the replicate trial plots for the four genotypes (in 2011) vs. day number in the year. The error bars are plus‐and‐minus one standard error of the means. The vertical lines show when the canopies first intercepted 90% or more of the incident PAR based on the LAIs and the fitted *k* values. The horizontal bar indicates June which has the peak in the annual PAR levels.

The leaf areas on each stem were then combined with the equivalent stem numbers m^−2^ to give the LAIs. Figure 4 shows the mean LAIs across the four replicate field plots of each genotype over the 2011 growing season. The maximum LAIs were in the order Gig‐311 > Goliath > Sac‐5 > Sin‐11. Gig‐311 achieved 90% PAR interception first, then Goliath and finally Sac‐5 and Sin‐11, and in all the genotypes, this point was reached after the peak PAR levels in June. This ordering was also reflected in the final above‐ground yields in January 2012 which using two‐way anova gave the following significantly different values (all in tonnes dry matter hectare^−1^): Gig‐311 15.27; Goliath 8.81; Sac‐5 4.97 and Sin‐11 4.34 (the last two were not significantly different). There were no significant block effects. The plateaux (steady state) LAIs on Fig. 4 were due to shading effects resulting in the death of lower stem leaves, whilst new leaves were still being produced.

### Calculation of the canopy extinction coefficients (*k*)

**Figure 5:**
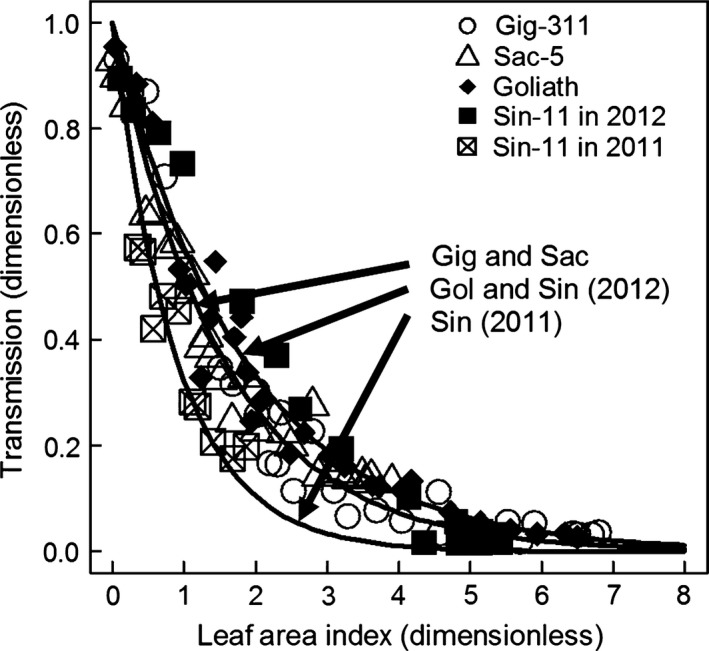
Transmission vs. leaf area index showing the three significantly different fitted lines used to estimate the extinction coefficients (*k*). Apart for Sin‐11, the 2011 and 2012 data have been combined. Thus, Sin‐11 has separate fits and *k* values for both years. For clarity, the standard errors are not shown.

The significantly different curves fitted to the transmission vs. LAI data are shown on Fig. 5 and the *k* values from the fits on Table 2. Gig‐311 and Sac‐5 have the same *k* value which is slightly larger than the value for Goliath and Sin‐11 (using Sin‐11's 2012 data only). Sin‐11 in 2011 had a very high *k* value, but by the following year, its *k* was the same as for Goliath.

**Table 2:** The significantly different canopy extinction coefficient values (*k*, m^2^ ground m^−2^ leaf) from the fitted lines shown in Fig. 5. The year values show the data sets combined to give the data fitted. Thus, only Sin‐11 had separate fits and hence *k* values for 2011 and 2012

Genotype	*k*‐Value	Standard error
Gig‐311 and Sac‐5 (both 2011 and 2012)	0.6539	0.01637
Goliath (2011 and 2012) and Sin‐11 (2012 only)	0.5533	0.01832
Sin‐11 (2011 only)	1.129	0.07686

### Leaf expansion rate (LER)

**Figure 6:**
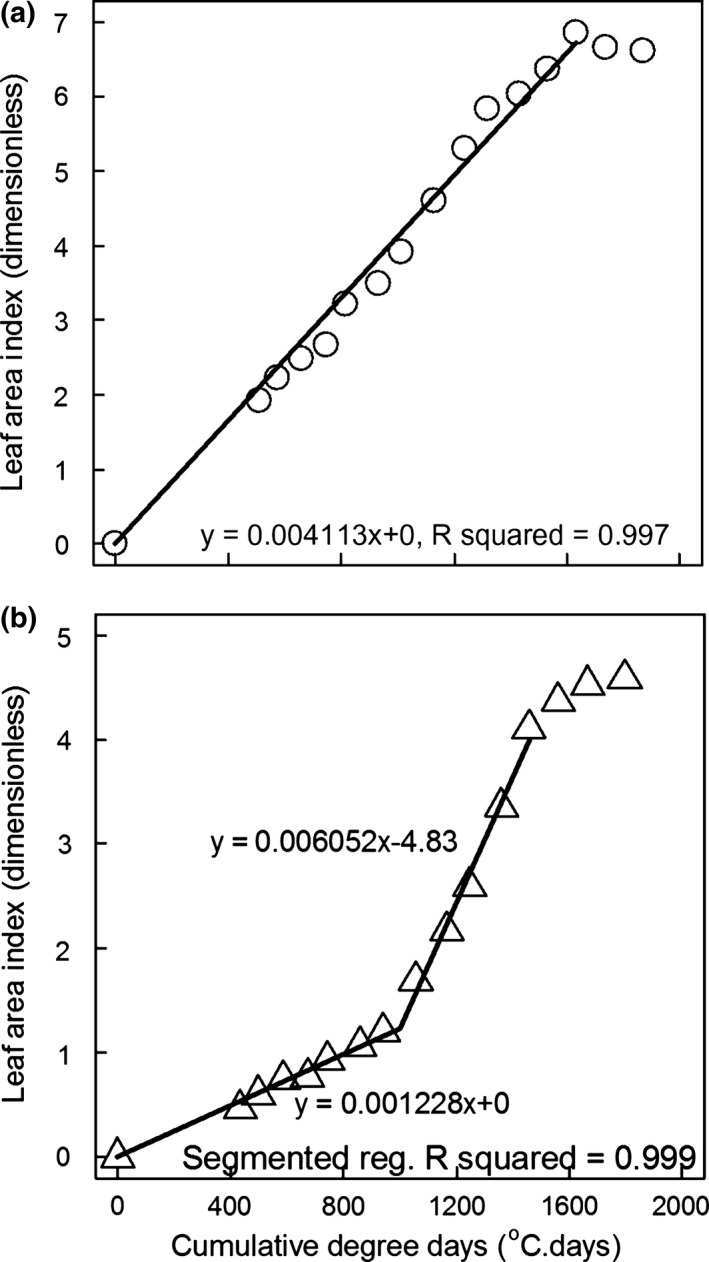
Measured LAI vs. cumulative degree days calculated using a *T*_b_ of 0 °C and with the LAI and degree days equal to zero on the day when the first leaves with ligules appeared (stage FLL day). Example data from individual trial plots are shown for two genotypes demonstrating the fits to the two models used: a single linear regression line ((a) Gig‐311) and segmented regression with two linear sections and one breakpoint ((b) Sac‐5). The slopes of the lines were used to estimate the LAI °C day^−1^ (leaf expansion rate: LER) values. The later season ‘plateau’ LAI values were not used in the fits.

Figure 6 shows examples of the LAI vs. cumulative degree days graphs used to estimate the LERs of the different growth phases. Example data showing the two methods used to fit straight lines to the data are shown (Fig. 6a, b). Two canopy expansion phases were identified; the initial phase 1 of growth was present in all the genotypes whilst Sac‐5 and Goliath showed the later second phase of LAI expansion. The mean LER values for each phase for each genotype after comparison by anova are given in Table 3. Only Sin‐11 and Goliath flowered in this trial. The former produced its flag leaves on day 196 whilst the latter did so on day 215. The secondary phase of growth and also the continued LAI increase of Sin‐11 even after flowering were due to a flush of new stems reaching the upper canopy and contributing to the leaf area capturing light.

**Table 3:** The significantly different leaf expansion rates (LER, LAI °C day^−1^) values (*T*_b_ = 0 °C) for the 1st and 2nd (later) phases of growth (in 2011). For genotypes with both growth phases, the breakpoint between the two phases is given as cumulative  °C days (*T*_b_ = 0 °C) after the 1st ligule leaves unfurled (see Table 1)

Genotype	Mean LAI °C day^−1^ for 1st growth phase	Std. error of mean LAI °C day^−1^ for 1st growth phase	Mean LAI °C day^−1^ for 2nd growth phase	Std. error of mean LAI °C day^−1^ for 2nd growth phase	Mean breakpoint cumulative °C day^−1^
Gig‐311	0.003931	0.0001141	–	–	–
Sac‐5	0.001395	0.00005638	0.006225	0.0003270	1029
Sin‐11	0.001395	0.00005638	–	–	–
Goliath	0.002276	0.0002752	0.006225	0.0003270	866

**Figure 7:**
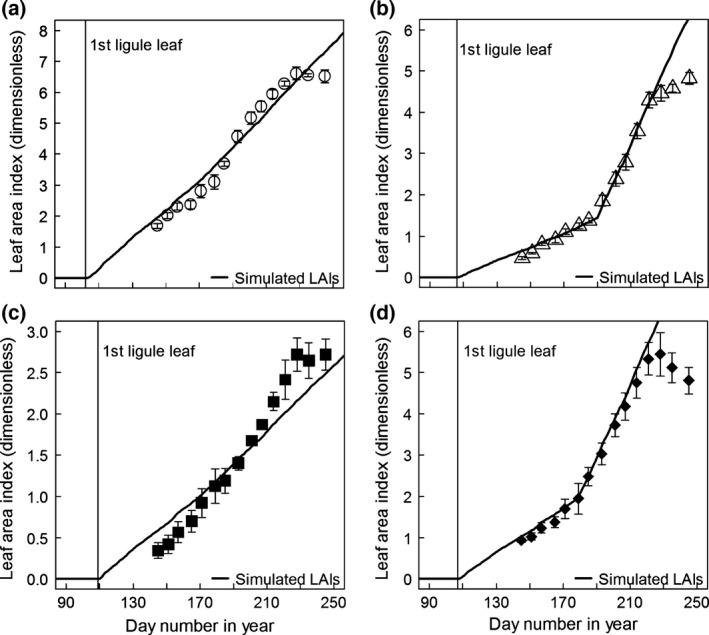
The mean LAIs across the replicate trial plots for the four genotypes vs. day number in the year in 2011: (a) Gig‐311, (b) Sac‐5, (c) Sin‐11 and (d) Goliath. The error bars are plus‐and‐minus one standard error of the mean. These are the data plotted on Fig. 4. The LAI values were assumed to be zero on the day when the first leaves with ligules appeared (stage FLL day, vertical lines on the plots). The lines through the data points are simulations with the model on Fig. 2 using the 2011 met data. The model parameterization for each genotype came from Tables 1, 2 and 3.

That the mean LER data in Table 3 would give a good estimate of the actual mean LAI data shown on Fig. 4 was checked on Fig. 7 by simulation. The simulated LAIs were a good fit to the data up to the plateaux (steady state) LAI points apart from Sin‐11 where the simulation was noticeably less consistent with the real data than for the other genotypes.

### Radiation‐use efficiencies (RUE)

The cumulative PAR estimates equivalent to the dry matter values from the destructive harvests were calculated as in the methods apart from for Sin‐11. The relatively poor simulation of LAI for this genotype warranted all the LAIs being derived by linear interpolation between the actual LAIs.

**Figure 8:**
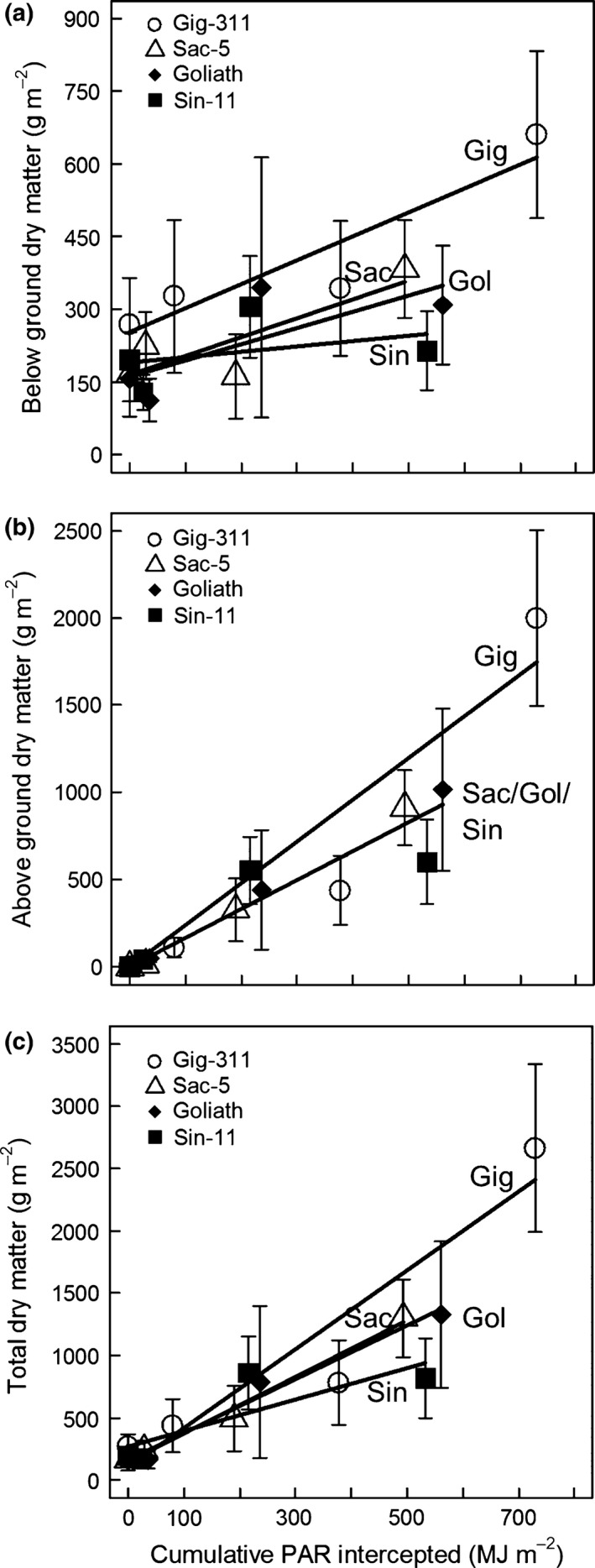
The dry matter accumulated by the plants vs. the cumulative PAR intercepted by their canopies as estimated by simulation and interpolation. The error bars are plus‐and‐minus one standard error of the mean dry matter values. (a) Below‐ground dry matter, (b) above‐ground and (c) total above‐ and below‐ground dry matter. The straight lines on (a) and (c) are linear regression fits to each genotype. The two significantly different lines on (b) are the minimal adequate model fitted by ancova. The slopes of the fitted lines gave the RUEs on Table 4.

Figure 8 shows the below‐ground, above‐ground and total dry matter vs. cumulative PAR interception for the four genotypes. Early season remobilization from the rhizome to support the start of the new season growth meant that the below‐ground data was expected to show physiological meaningful deviations from linearity. Hence the below ground and total biomass were tentatively fitted using linear regression to give the RUEs (slopes) in Table 4. The above‐ground data were expected to increase reliably with increased PAR interception (Beale & Long, 1995; Clifton‐Brown *et al*., 2000) and so were analysed using ancova (see Table 4). This showed that the above‐ground RUE for Gig‐311 was 2.40 gDM MJ^−1^ PAR intercepted and was significantly higher than the other three genotypes which were not significantly different from each other with 1.66 gDM MJ^−1^ PAR. Thus, Gig‐311's above‐ground RUE is 45% higher than the other genotypes. The data may also indicate that the early flowering of Sin‐11 might have reduced its yield in line with the findings of Jensen *et al*. (2013). The cumulative PAR in the model in Fig. 2 can be converted to dry matter yield by direct multiplication by the RUE. Therefore, any increases in RUE would give directly proportional increases in yield.

**Table 4:** The RUEs estimated from the slopes of the linear regression or ancova fits to the data in Fig. 8 (in 2011)

Genotype	Biomass used in fit	Slope (RUE) gDM MJ^−1^ PAR	y‐intercept gDM m^−2^	*R* ^2^
Gig‐311	Below ground	0.49	253.04	0.86
	Above ground^a^	2.40	0.00	
	Total	3.16	99.26	0.90
Sac‐5	Below ground	0.39	164.65	0.72
	Above ground^a^	1.66	0.00	
	Total	2.27	143.89	0.99
Sin‐11	Below ground	0.11	189.66	0.14
	Above ground^a^	1.66	0.00	
	Total	1.26	265.75	0.68
Goliath	Below ground	0.34	160.86	0.58
	Above ground^a^	1.66	0.00	
	Total	2.16	157.90	0.98

a From minimal adequate model fitted using ancova (*R* ^2^ of fit: 0.94).

### Simulations

**Figure 9:**
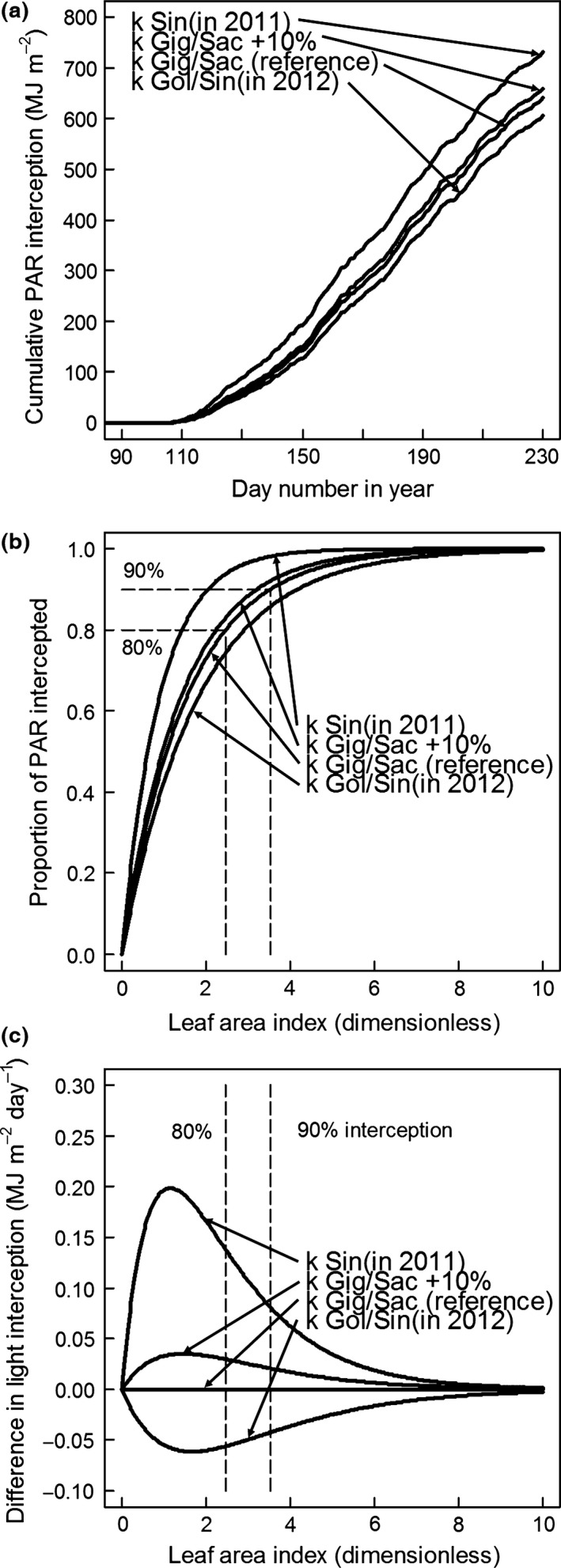
(a) The effect of changes in canopy extinction coefficient (*k*) of Gig‐311 on cumulative PAR interception vs. day number. The plots were produced by simulation starting at the stage FLL day using the model on Fig. 2 and parameterized using the values for Gig‐311 on Tables 1, 2 and 3. The reference line was for Gig‐311 using its *k* value. Also shown are the effects on cumulative PAR interception of increasing Gig‐311's *k* value by 10% and from using the *k* values for Goliath/Sin‐11 (in 2012) and Sin‐11 (in 2011) from Table 2 (all other parameterization remained the same as for the reference simulation). Note that Sac‐5 has the same *k* value as Gig‐311. (b) The proportion of PAR intercepted by the canopy vs. LAI for the *k* values used in (a). The dashed lines show when Gig‐311 achieves 80% and 90% interception. (c) At each of the LAIs and *k* values in (b), the proportion of PAR intercepted was used to calculate the daily PAR interception if the canopies had been illuminated with an intensity of 1 MJ PAR m^−2^ ground day^−1^. These interceptions were then expressed as differences relative to that of Gig‐311 with its own *k* value (reference lines on (a), (b) and (c)) and plotted vs. LAI.

Figure 9a shows the effect on the cumulative PAR interception of Gig‐311 (reference simulation) of changing its *k* value to that of the other genotypes or of increasing its *k* value by 10%. Although the Sin‐11 *k* value in 2011 (1.13) was 73% higher than Gig‐311's *k* value (0.65) when the Gig‐311 simulation was run using this value, it only gave a 14.1% increase in cumulative PAR by day 230. Likewise, a 10% increase in Gig‐311's *k* only gave a 2.9% increase in the day 230 cumulative PAR compared to the reference simulation.

Figure 9b plots the proportion of PAR intercepted vs. LAI for the *k* values used in Fig. 9a. Gig‐311 can intercept 90% of the incident PAR at the fairly low LAI of 3.5 (Clifton‐Brown *et al*., 2000) which it reached at the beginning of July in 2011 (Fig. 4). By the end of that growth season, Gig‐311 had almost doubled this LAI. The 90% interception dates are shown for each genotype on Fig. 4.

Increases in *k* made surprisingly modest changes in the cumulative PAR interception because a 10% increase in *k* (relative to the *k* of Gig‐311) only gives a 10% increase in the light interception as LAI approaches 0: when the leaf area is so small that virtually no light is intercepted. As the LAI increases the percentage, increase in interception rapidly falls and even at quite low LAIs essentially all the light is intercepted (see Fig. 9b) and so there is then no difference in PAR captured. This point is made clearer if the PAR intercepted over a day, which gives the daily yield, is considered. Figure 9c shows the changes in the (normalized daily) PAR interception ability of a *Miscanthus* canopy relative to Gig‐311's at a given LAI caused by changes in *k* value. Only over a restricted range of quite low LAI values does an increase in *k* allow the canopy to outperform Gig‐311's at the same LAI, and then, the increase is lower than the percentage rise in *k* value. For instance, on Fig. 9c, when Gig‐311 is intercepting 80% of the light (e.g. 0.80 MJ PAR m^−2^ day^−1^ if the incident radiation is 1.0 MJ PAR m^−2^ day^−1^), a canopy with the *k* value of Sin‐11 in 2011 would only be collecting an additional 0.14 MJ PAR m^−2^ day^−1^ despite the 73% higher *k* value. In any case, the low LAIs that give the peak increase in canopy performance are exceeded by Gig‐311 early in the growing season and it gets to those levels far sooner than the other genotypes (Fig. 4).

**Figure 10:**
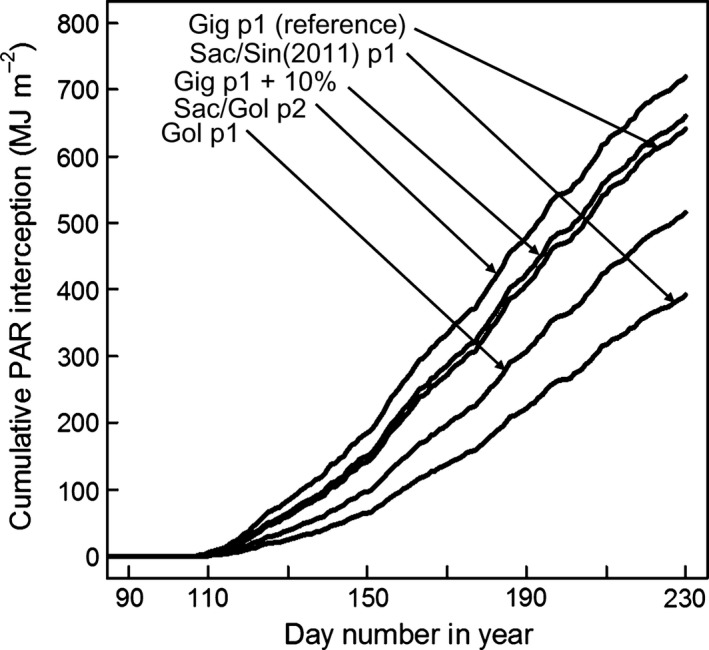
The effect of changes in LER (LAI °C day^−1^) on the cumulative PAR interception of Gig‐311 vs. day number. The plots were produced by simulation using the model on Fig. 2 and parameterized using the values for Gig‐311 on Tables 1, 2 and 3 (starting at the stage FLL day). The reference line was for Gig‐311 using its actual LAI °C day^−1^ value for growth phase 1 (p1 on figure). Also shown are the effects on the cumulative PAR interception of increasing Gig‐311's value by 10% and from using the LAI °C day^−1^ values for phase 1 Sac‐5/Sin‐11 (in 2011); Goliath phase 1; and Sac‐5/Goliath phase 2 (p2 on figure). The LER values were from Table 3, but all other parameterization remained the same as for the reference simulation.

There are two aspects to the rate of canopy expansion: the leaf expansion rate (LER) in LAI °C day^−1^ and the actual rate of canopy expansion on a given day in LAI increase day^−1^ (▵LAI_t_ on Fig. 2) which results from multiplying the degree days contributing to canopy expansion by LER. The magnitude of the degree day contribution on a given day is in turn controlled by the base temperature for canopy expansion (*T*_b_). For Gig‐311, *T*_b_ is thought to be 10 °C (Clifton‐Brown *et al*., 2000) although values as low as 6°C have also been estimated for *Miscanthus* (Price *et al*., 2003; Farrell *et al*., 2006). The potential effect of decreasing *T*_b_ from 10 to 6 °C on degree days can be seen on the temperature profile above these temperatures on Fig. 3. The effect of changes in leaf expansion rate (LER) on cumulative PAR is shown on Fig. 10. A 10% increase in Gig‐311's LAI °C day^−1^ only gave a 2.9% increase in the cumulative PAR intercepted by day 230.

**Figure 11:**
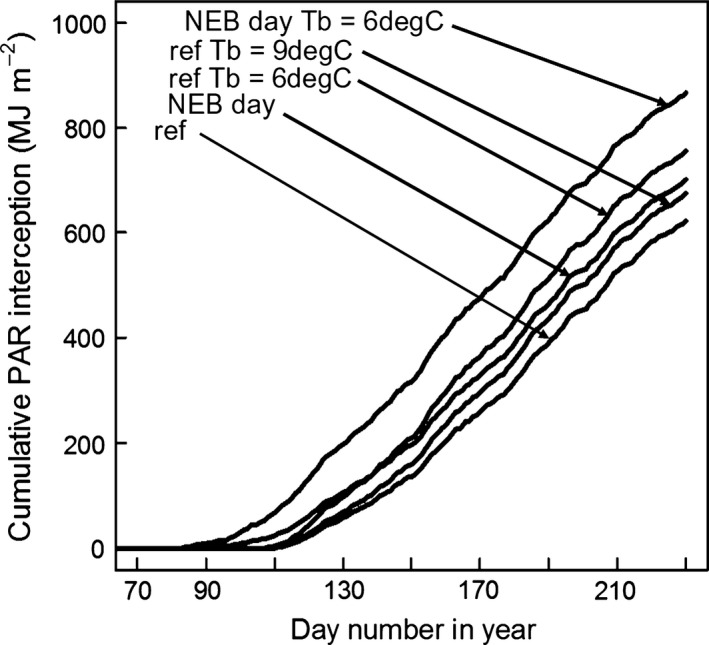
The effect of changes in the start day for canopy expansion and of changes in base temperature for canopy expansion (*T*_b_) on the cumulative PAR interception of Gig‐311. The plots were produced by simulation using the model on Fig. 2 but with a *T*_b_ of 10 °C and its equivalent fitted LAI °C day^−1^ for Gig‐311 of 0.01668. The other parameters were for Gig‐311 from Tables 1 and 2. The reference line is for Gig‐311 starting at the actual day when the canopy started to expand (stage FLL). The effect of decreasing the *T*_b_ value by 10% to 9 °C and to 6 °C but keeping all the remaining reference line parameterization the same are shown. The impact on the cumulative PAR interception of allowing the canopy to start expanding from the day when the buds first emerged (stage NEB) is also illustrated. Two such simulations are shown as follows: the first with the reference line parameterization but starting at the bud emergence day, and the second doing the same but with a *T*_b_ of 6 °C as well. The former shows that the leaf production should have been possible earlier in the year than was observed in the field.

The simulations on Fig. 11 use Gig‐311's actual base temperature for canopy expansion (*T*_b_) of 10 °C. The reference simulation is for Gig‐311 using the *T*_b_ of 10 °C and starting at the actual start day for canopy expansion (stage FLL day). Shown are the effects on cumulative PAR interception of reducing the *T*_b_ by 10% to 9 °C and then to 6 °C. The effect on the reference simulation of allowing canopy expansion as soon as the buds appeared (stage NEB day) and of also doing this with a *T*_b_ of 6 °C is also shown. The percentage increases in cumulative PAR intercepted by day 230 relative to the reference simulation are as follows: *T*_b_ of 9 °C 8.1%, *T*_b_ of 6 °C 21.1%, reference starting at NEB day 12.4% and the NEB day start with *T*_b_ 6°C 39.0%. The simulation starting at the NEB day with a *T*_b_ of 10 °C had a LAI of 0.48 by the real day on which the canopy started to expand (stage FLL day). This LAI is some fifteen times larger than that given by the actual leaf areas of all the first ligule leaves and indicates inhibition of leaf production early in the growing season.

### Replicated trial data

Yield data collected from the replicated Rothamsted Research trial were compared to the results from the model. The model was run using the meteorological and emergence data collected from the Rothamsted trial. The model over predicted yield compared to the observed data in both 2011 and 2012. A potential cause for the reduction in yield was drought; however, attempts to introduce drought into the model by reducing RUE with respect to soil moisture deficit proved ineffective, but did reduce the error. This implies that other factors are effecting the yield at Rothamsted. A reduction in LER due to droughting (Clifton‐Brown *et al*., 2000) is a likely contributor, but there are also possible differences in crop maturity between the sites due to their different climates.

## Discussion

### Effect of the canopy extinction coefficient *k* on cumulative PAR interception

Canopy architecture is crucial to intercepting light and hence producing yield. Increases in the canopy extinction coefficient (*k*) should result in higher cumulative PAR interception and hence give a larger yield. This study used genotypes with the extremes of *Miscanthus* canopy architecture and estimated their *k* values. The Gig‐311 *k* value of 0.65 is very close to the 0.68 previously found for this genotype in Clifton‐Brown *et al*. (2000). However, Cosentino *et al*. (2007) found a *k* of 0.56 for *M*. × *giganteus* which is close to the values for the sinensis types (Goliath and Sin‐11) found here. Vargas *et al*. (2002) gave the Goliath *k* value as 0.66. Thus, despite the large differences in canopy architecture, the *k* values are very similar. The exception in this study was for Sin‐11 in 2011 which may have still been immature in that year as its high *k* value became very similar to the other genotypes in the following year. Sin‐11 matured more slowly than the other genotypes due to slower growth rates, which delayed canopy closure in Aberystwyth at the planting density of 2 m^−2^. The high extinction coefficient does suggest that the genetic flexibility for high *k* exists in *Miscanthus* and that stand maturity may affect it in some genotypes.

Gig‐311 is a superior performing genotype (Naidu *et al*., 2003; Wang *et al*., 2008; Dohleman & Long, 2009; Dohleman *et al*., 2009) and currently the only one commercially available. Therefore, the aim of breeding research is to outperform *M*. × *giganteus* (of which Gig‐311 is an example). The effect on cumulative PAR interception of introducing the *k* values observed in Sin‐11 and Goliath into Gig‐311 to simulate the creation of a new hybrid made surprisingly modest changes in PAR interception.

The time period in which increases in the *k* value had the greatest impact on light interception was at the beginning of the growing season before LAI ≥4. After this stage, most of the light is intercepted in all genotypes and so increases in *k* had little effect. Therefore, to exceed the performance of Gig‐311, the duration of maximum canopy interception must be extended beyond the current length of the growing season. Peak PAR levels at IBERS are in June, and by September, light levels are already approximately the same as March. Thus, there is considerable under‐utilized PAR in the spring when the canopies are still developing. However, temperatures in this period are usually still cold which is inhibitory to growth, risks destruction of the early canopy from frost and could reduce RUE (Hastings *et al*., 2009b) and so breeding for cold tolerance is important. In the warmer climate of south‐east England (compared to IBERS), Beale & Long (1995) found that *M*. × *giganteus* achieved 90% light interception in early June, whilst the cooler conditions at IBERS meant that the June PAR peak was nonoptimally intercepted. This emphasizes the need to include the effect of temperature on canopy expansion in any consideration of light interception. To accelerate canopy closure either stem emergence along with the start of canopy expansion must be brought forward and/or the early spring canopy growth rate must be increased.

### Emergence and the start and rate of canopy expansion

The base temperature for canopy expansion (*T*_b_), photoperiod or a combination of these with a threshold level of accumulated degree days has been thought to control when new buds first appear above ground at the start of a growing season (stage NEB day) (Clifton‐Brown *et al*., 2000; Farrell *et al*., 2006; Hastings *et al*., 2009b). The emergence of Gig‐311 in this study is consistent with the 12‐h photoperiod emergence criteria used Hastings *et al*. (2009b). However, if the temperature of the rhizome controls emergence of new buds/stems, then the *T*_b_ of 10 °C is too high as we observed stem emergence at temperatures below this value. We also observed that the new buds of each of the genotypes emerged at different times and the processes controlling this and regreening from cut stems are unknown. The wide geographic distribution of *Miscanthus* may mean that different genotypes have different emergence mechanisms attuned to the requirements of their original locations.

For Gig‐311 and Sac‐5, the emergence of the stems did not coincide with the production of unfurled leaves with ligules (see also Beale & Long, 1995) even though the temperatures were conducive to leaf production in Gig‐311. The young buds of Gig‐311 are particularly susceptible to frost damage and yet its stems appeared above ground well before its canopy started to expand (Zub *et al*., 2012). Frost damage to the stems would result in more resources being drawn from the rhizome to restart growth and repeated episodes could eventually kill the plants with insufficient rhizome resources to drive another flush of shoots. Inhibition of leaf production could be due to photoperiod, temperature or accumulated degree day thresholds or to cold stress in the mornings inhibiting leaf expansion later in the day.

Cumulative PAR interception was not very sensitive to increases in LER over Gig‐311's already high rate. The role of the *T*_b_ value in controlling the canopy expansion, especially in the early part of the growing season, is complex. If the daily average air temperatures are about equal to *T*_b_, then some days will not contribute degree days whilst many others only contribute for part of the day. If the *T*_b_ is lowered, then substantial increases in the daily degree day contributions are possible in the spring. The higher degree day values would result in the LAI expanding rapidly with concomitant substantial increases in early season PAR interception.

The large genetic variability in *Miscanthus T*_b_ values combined with the potential gains in yield that lowering it could make means this is a good breeding target especially if the start of canopy expansion could be moved to earlier in the season. The beneficial impact of early canopy establishment on yield has been demonstrated in a study of 244 *Miscanthus* genotypes (Robson *et al*., 2013). A lower *T*_b_ than Gig‐311's might be less beneficial in a warmer climate where the canopies would reach levels intercepting most of the light earlier in the year anyway. In addition, to gain the full advantage from a lower *T*_b_, the LER should not decrease appreciably (Farrell *et al*., 2006). Increases in LER could have useful synergistic effects when combined with a lower *T*_b_. To be useful in a breeding programme, simple and quick methods need to be developed to measure these traits and their narrow sense heritabilities (h^2^) in the actual breeding populations needs to be sufficiently high to make them useful for selection (Falconer, 1989).

### Radiation‐use efficiencies (RUE)

The PAR accumulated over a growing season is not the only factor dictating final yield, the efficiency with which that energy has been converted to dry matter (i.e. RUE) is also important. For *Miscanthus* where the above‐ground material is harvested, it is the above‐ground RUE that is most important. For Gig‐311, this was estimated as 2.40 g dry matter MJ^−1^ PAR intercepted which was close to the 2.35 gDM MJ^−1^ found by Clifton‐Brown *et al*. (2000) in Ireland. The Gig‐311 above‐ground RUE was 45% higher than for the other three genotypes (which included both its nominal parent species) which in part ex*plains* why it is such a productive hybrid. The physiological mechanism causing Gig‐311's RUE being higher than the other genotypes in this trial is not known although heterosis is the likely genetic cause.

RUE estimated using harvested dry matter is net of the photosynthetic rate offset by many factors that remove dry matter after photosynthesis has created it. Thus, it is not surprising that there is variation in the RUE values for *Miscanthus* in the literature (Beale & Long, 1995; Clifton‐Brown *et al*., 2000; Zub & Brancourt‐Hulmel, 2010; Kiniry *et al*., 2012). Such variation in RUE is also a complicating factor in studies attempting to relate canopy duration with yield (Robson *et al*., 2013). The RUE estimated here of 2.40 gDM MJ^−1^ is much lower than the 3.7 gDM MJ^−1^ found for *M*. × *giganteus* in a trial in the USA (Kiniry *et al*., 2012). The probable cause of this is the comparatively cool summers in West Wales (U.K.) which would limit biomass accumulation and the RUEs seen. Thus, the RUE values in this paper are strictly only applicable to areas of similar climate and probably explains why the value for *M*. × *giganteus* grown in Ireland (Clifton‐Brown *et al*., 2000) is so close to that in Wales. Some biofuel crops such as switchgrass can have very high RUEs of 4–5 gDM MJ^−1^ under favourable climates (Kiniry *et al*., 1999). This emphasizes the need for breeding for increased biomass and RUE in *Miscanthus* if full advantage is to be taken of its ability to grow on marginal land with low agricultural inputs.

### Work for the next loop of the modelling cycle

From modelling *Miscanthus* yield *across* genotypes using the model in this manuscript and using a more detailed model as well, it became clear that several processes important in yield production are far from well understood. The current understanding of emergence failed to predict the field outcomes and even the emergence of *M*. × *giganteus* needs clarification. Despite evidence that flowering decreases yield in *Miscanthus* (Jensen *et al*., 2013), there is no comprehensive understanding of what triggers flowering or its potential impact on RUE and senescence. Senescence processes other than those triggered by frost are also in need of further investigation. Any potential effect of plant maturity on yield could not be modelled and meaningfully parameterized in a short‐term trial such as this one, but the fact that the estimated *k* and RUE values for *M*. × *giganteus* were so close to those previously measured on older stands (Clifton‐Brown *et al*., 2000) suggests that these key model parameters do not vary much with maturity (unless the plants are very immature). Thus, for future progress towards detailed models that can incorporate the diversity seen in *Miscanthus*, research is needed to produce full processes descriptions of several key stages in its growth and development which are based on solid physiological knowledge. Experiments are then needed under controlled (and field) conditions to parameterize these process descriptions.

In summary, this study has demonstrated that extending canopy duration at the start of the growing season has the potential to increase yields to a greater extent than improving *k* (i.e. changing canopy architecture). From the results, it is clear that Gig‐311 is such a high yielding hybrid (under nondrought conditions) because it has a slightly higher *k* value, more rapid leaf expansion and significantly higher above‐ground RUE compared to the other genotypes studied. If a frost resistant hybrid could be bred that combined the high RUE and *k* of Gig‐311 with a lower *T*_b_ and earlier canopy expansion, then significant increases in yield are achievable.
